# Box–Behnken Optimization of Soybean Meal Enzymatic Digestion for Small-Peptide Production

**DOI:** 10.3390/foods15030474

**Published:** 2026-01-29

**Authors:** Xiao Zhang, Qixuan He, Junmei Li, Yan Zhang, Jiang Yuan, Changjiang Zang, Fengming Li

**Affiliations:** 1 College of Animal Science, Xinjiang Agricultural University, Urumqi 830052, China; 18139230168@163.com (X.Z.); 320232645@xjau.edu.cn (Q.H.); 15348010072@163.com (J.L.); 15026300105@163.com (Y.Z.); 18999361205@189.cn (J.Y.); zcj780@126.com (C.Z.); 2 Xinjiang Taikun Group Co., Ltd., Changji 831100, China

**Keywords:** soybean meal, enzymatic digestion, response surface, Fourier transform infrared spectroscopy (FTIR)

## Abstract

This study used soybean meal as the substrate and systematically optimized its enzymatic hydrolysis through single-factor experiments and response surface methodology. A predictive model based on a Box–Behnken design was developed to improve protein hydrolysis efficiency and increase the yield of functional products. The optimal conditions were 1.45% enzyme addition, a reaction time of 62 h, a temperature of 36.5 °C, and a moisture content of 35%. Under these conditions, the small-peptide content increased 16.33-fold. Structural analyses showed that enzymatic treatment markedly disrupted the compact surface of soybean meal, converting it into a loose, porous matrix. In addition, enzymolysis altered the protein secondary structure from ordered α-helices and folded conformations to more disordered, flexible forms, thereby improving the molecular-weight distribution. Composition analyses showed an 114.2% increase in total free amino acids, including essential amino acids. Moreover, DPPH radical-scavenging activity increased from 18.37% to 57.99%. Overall, this study optimized the enzymatic hydrolysis conditions for soybean meal and provides valuable insights for the development of high-value protein-peptide products.

## 1. Introduction

Soybean meal (SBM) is widely recognized as the primary plant-based protein source in global livestock production because of its high protein content and balanced amino acid profile [1]. However, modern high-density, antibiotic-free production systems pose substantial challenges to intestinal health and feed efficiency. Unprocessed SBM contains several antinutritional factors—such as trypsin inhibitors, antigenic proteins, and oligosaccharides—that impair protein digestion, trigger intestinal immune stress, and disrupt the intestinal barrier. These limitations collectively constrain the effective use of SBM in animal husbandry [2,3,4].

To improve the nutritional quality of SBM and reduce its antinutritional components, enzymatic hydrolysis has become a major focus of research in feed science. This process effectively degrades large proteins into small peptides and free amino acids—particularly dipeptides and tripeptides—which represent the most efficiently absorbed forms [5]. These low-molecular-weight products are rapidly transported across intestinal cells via specific peptide transporters, resulting in higher bioavailability than free amino acids. In addition, enzymatic treatment substantially decreases antinutritional factors and produces bioactive peptides with antioxidant and immunomodulatory functions [6], both of which are important for reducing antibiotic use and promoting animal health. However, the efficiency of enzymatic hydrolysis is influenced by multiple parameters, including enzyme type, concentration, temperature, pH, and reaction time. The interactions among these variables create a complex optimization landscape. Conventional one-factor-at-a-time approaches are time-consuming and often fail to capture potential synergistic effects. In contrast, response surface methodology offers an effective strategy by using statistical modeling to simultaneously evaluate multivariable interactions and identify optimal processing conditions with fewer experimental trials [7].

This study systematically optimized the enzymatic digestion of SBM using response surface methodology to increase the yield of small peptides. A mathematical model was further developed to quantify the relationship between processing parameters and small-peptide content, enabling the prediction of optimal hydrolysis conditions. The findings aim to provide a scientific basis and technical support for the development of highly digestible, functional SBM-derived peptide feed ingredients. Compared with previous SBM enzymatic optimization studies, the novelty of this work lies in incorporating moisture content alongside enzyme dosage, temperature, and time in a four-factor Box–Behnken design, thereby better reflecting practical semi-solid processing conditions. In addition, we go beyond parameter optimization by integrating SEM, FTIR-based secondary structure analysis, molecular weight distribution, and free amino acid profiling to mechanistically link processing conditions to small-peptide generation and product quality.

## 2. Materials and Methods

### 2.1. Materials

Soybean meal (CP ≥ 46%; Xinjiang Taikun Inc., Changji, China). The protease used in this study (60,000 U/g; Hebei Haotong Biotechnology Inc., Cangzhou, China) is a *Bacillus*-fermentation–derived composite protease, characterized by a slightly alkaline optimum (optimum temperature: 40 °C; optimum pH: 7.2). Detailed enzyme composition (e.g., proportions of alkaline/neutral proteases) was not specified by the manufacturer; therefore, key activity parameters are reported here to ensure reproducibility.

### 2.2. Preparation of Enzymatic Protein Feed

Prior to hydrolysis, the soybean meal (SBM) slurry pH was adjusted to 7.2, and pH was monitored during hydrolysis to ensure it remained within the protease complex’s effective pH range. A total of 500 g of SBM was weighed into a beaker, and an appropriate amount of water and the protease complex was added. The moisture content was adjusted from 25% to 45% in 5% increments, corresponding to an SBM (dry matter)-to-added-water ratio (*w*/*w*) of 1:0.33–1:0.82 (i.e., 1:0.33, 1:0.43, 1:0.54, 1:0.67, and 1:0.82 for 25%, 30%, 35%, 40%, and 45% moisture, respectively). The mixture was stirred continuously with a glass rod until thoroughly homogenized, and then allowed to stand. Enzymatic hydrolysis was performed as a semi-solid-state fermentation (semi-solid-state hydrolysis), in which the substrate was adjusted to the target moisture content to form a paste-like matrix rather than a free-flowing liquid system; therefore, rpm-controlled mechanical agitation was not applied. Three parallel replicates were prepared for each treatment. Enzymatic hydrolysis was carried out in a constant-temperature incubator. After hydrolysis, the samples were dried to constant weight at 65 °C, ground, and stored at −20 °C for analysis.

### 2.3. Single-Factor Experiments

SBM served as the substrate for enzymatic hydrolysis. Four parameters—enzyme addition, hydrolysis temperature, hydrolysis time, and moisture content (Factor ranges were determined from preliminary trials, the literature, and semi-solid processing feasibility)—were evaluated for their effects on water-soluble protein and small-peptide contents. Enzyme addition levels of 0.5%, 1.0%, 1.5%, 2.0%, and 2.5% were tested. Hydrolysis temperatures were set at 30, 35, and 40 °C. Hydrolysis times were 24, 48, and 72 h. Moisture content was adjusted from 25% to 45% in 5% increments. Although pH is a critical determinant of protease activity, it was not treated as a variable in the single-factor experiments because the initial pH was adjusted to the reported optimum (pH 7.2) for the protease complex and was monitored during hydrolysis to maintain stable reaction conditions. Therefore, the single-factor screening focused on process parameters (enzyme addition, time, temperature, and moisture content) that are most relevant to semi-solid operation and subsequent Box–Behnken optimization.

### 2.4. Optimization of Response Surface

Based on the results of the single-factor experiments, four factors—enzyme addition, hydrolysis temperature, hydrolysis time, and moisture content—were selected for further optimization. Response surface analysis was performed using a Box–Behnken design with four factors and three levels, implemented in Design-Expert 13.0.1 (Stat-Ease Inc., Minneapolis, MN, USA). Experimental runs were conducted according to the combinations generated by the software, and the corresponding data were collected. Statistical significance was set at *p* < 0.05. The experimental data were then subjected to analysis of variance and quadratic regression to construct a predictive model that describes the effects of linear, interaction, and quadratic terms. This model was used to evaluate relationships among the factors and small-peptide content and to identify the optimal hydrolysis conditions.

### 2.5. Measurement of Water-Soluble Protein Levels

Water-soluble protein content was determined using the method described by Fu Sheng Chen et al. [8]. In brief, 0.1 g of enzymatic hydrolysate powder was placed in a 250 mL conical flask; then, 40 mL of distilled water was added, and the mixture was vortexed. The mixture was centrifuged at 2750× *g* for 5 min. Subsequently, 100 μL of the resulting supernatant was transferred to a tube, mixed with 5 mL of Coomassie Brilliant Blue solution, vortexed, and incubated at room temperature in the dark for 2 min. Absorbance was immediately measured at 595 nm using a UV-1800 spectrophotometer (Shimadzu Inc., Kyoto, Japan). A bovine serum albumin standard curve was generated with the regression equation y = 0.0365x + 0.5543 (R^2^ = 0.9993). Water-soluble protein content (mg/g) was calculated from the standard curve.

### 2.6. Identification of Free Amino Acids

A total of 1.0 g of sample was placed in a 50 mL volumetric flask, dissolved in water, and mixed thoroughly. The mixture was allowed to stand at 4 °C for 24 h. Then, 2 mL of the supernatant was transferred to a 50 mL centrifuge tube, mixed with an equal volume of 5% sulfosalicylic acid, and centrifuged at 6000× *g* for 10 min. A 2 mL aliquot of the resulting supernatant was concentrated with a rotary evaporator (RE-52). The concentrate was dissolved in 1 mL of sodium citrate buffer and filtered through a 0.45 μm membrane. Free amino acids were quantified with a Biochrom 30+ automatic amino acid analyzer (Biochrom Inc., Cambridge, UK).

### 2.7. DPPH Free Radical Scavenging Assay

Weigh 0.1 g of the sample, add 1 mL of 80% methanol to achieve a 1:10 (g/mL) ratio, homogenize in an ice-water bath, and then centrifuge at 12,000 r/min for 10 min. Use the supernatant to determine DPPH free radical-scavenging activity according to the kit instructions. For measurement, add 400 μL of the sample solution to a test tube; then, add 600 μL of the DPPH working solution provided in the kit. Set up a sample control tube (400 μL of sample solution + 600 μL of 80% methanol) and a blank control tube (400 μL of 80% methanol + 600 μL of working solution). Mix thoroughly and incubate in the dark at 25 °C for 30 min. After centrifuging at 4000 r/min for 5 min, transfer 800 μL of the supernatant to a 1 cm optical path cuvette. Zero the spectrophotometer with 80% methanol; then, measure the absorbance at 517 nm. Each sample is tested in triplicate. Use the Trolox standard provided by the kit to prepare a concentration gradient from 0 μg/mL to 25 μg/mL in 80% methanol, and perform the same procedure to create a standard curve. The DPPH free radical scavenging rate is calculated using the following formula:

$$DPPH\;scavening\;activity\;(\%)=\left[1-\frac{\left(A_{s}-A_{sb}\right)}{A_{C}}\right]\times 100$$

*A_S_*: experimental group; *A_sb_*: control group; *A_C_*: blank control.

### 2.8. Scanning Electron Microscopy

Scanning electron microscopy (SEM) was used to examine the microstructure of soybean meal (SBM) before and after enzymatic hydrolysis. Samples were mounted on SEM stubs and sputter-coated with a thin gold layer using a vacuum sputter-coating system. Microstructural images were acquired with a Hitachi SU-8600 scanning electron microscope (Hitachi High-Tech Corporation, Tokyo, Japan) operating at 5.0 kV. When applicable, elemental analysis was performed using an energy-dispersive X-ray spectroscopy (EDS) detector (Oxford Ultimax 40, Oxford Instruments NanoAnalysis, Abingdon, UK).

### 2.9. Determining the Secondary Structure of Proteins

Spectral scanning of the samples was performed using a Thermo Fisher Nicolet iS50 Fourier transform infrared (FTIR) (Thermo Fisher Scientific, Waltham, MA, USA). spectrometer. The scan resolution was set to 4 cm^−1^, and spectra were collected over 800–4000 cm^−1^. Each sample was scanned 32 times, and the system automatically averaged the scans to improve the signal-to-noise ratio. The spectra used for secondary-structure analysis are representative averages, saved in CSV format. Subsequently, the spectra were imported into PeakFit 4.1.2 for calibration and deconvolution, and graphs were created in Origin 2024 (OriginLab Inc., Northampton, MA, USA) to analyze the protein secondary structure.

### 2.10. Estimation of Protein Relative Molecular Weight

The protein molecular-weight distribution was analyzed using a Waters 2695 high-performance liquid chromatography (HPLC) system (Waters Inc., Milford, MA, USA) equipped with a 487 UV detector and Empower GPC 3 workstation software. The chromatographic conditions were as follows: TSKgel 2000SWxl column (Tosoh Bioscience, Tokyo, Japan) (300 mm × 7.8 mm), mobile phase of acetonitrile/water/trifluoroacetic acid (40:60:0.1, *v*/*v*), UV detection at 220 nm, flow rate of 0.5 mL/min, and column temperature of 30 °C. The SEC-HPLC molecular-weight (MW) calibration curve was established using the following standards: cytochrome c (12,384 Da), aprotinin (6500 Da), bacitracin (1422 Da), Ala–Ala–Tyr–Arg (451 Da), and Ala–Ala–Ala (189 Da). All standards were analyzed under the same chromatographic conditions as the samples, and the calibration curve was constructed by plotting log(MW) versus retention time (t_R). The MW distribution of the hydrolysates was derived from the calibration curve. In this manuscript, “small peptides” are operationally defined as peptide fractions with molecular weights <2000 Da (i.e., 180–2000 Da). The small-peptide content was quantified throughout the study as the percentage of the SEC-HPLC peak area within this MW range, based on the established calibration curve.

### 2.11. Statistical Analysis

Free amino acid data were analyzed in SPSS 19.0 (SPSS Inc., USA) using one-way ANOVA and paired-sample *t*-tests. Results were reported as mean ± standard deviation. Statistical significance was set at *p* < 0.05, and high significance at *p* < 0.01. Data processing and plotting were performed in Origin 2024 (OriginLab Inc., USA).

## 3. Results

### 3.1. Single-Factor Experiments Results

**Figure 1:**
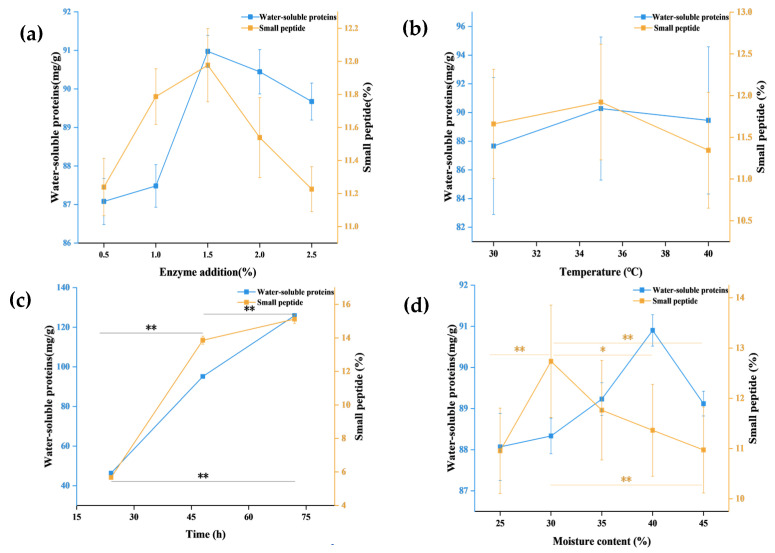
(**a**) Effect of enzyme addition amount on water-soluble protein and small-peptide content. (**b**) Effect of enzymatic temperature on water-soluble protein and small-peptide content. (**c**) Effect of enzymatic time on water-soluble protein and small-peptide content. (**d**) Effect of moisture on water-soluble protein and small-peptide content. (As shown in the figure, black lines indicate significant differences for both response variables, whereas a single-colored line indicates significance for only one response variable. Asterisks denote significance levels, with ** indicating *p* < 0.01 and * indicating *p* < 0.05.)

Figure 1a shows that when hydrolysis time, moisture content, and temperature are held constant, water-soluble protein and small-peptide levels in SBM initially increase and then decrease with increasing enzyme addition. The maximum values were observed at 1.5% enzyme addition, reaching 90.97 ± 2.93 mg/g for water-soluble protein and 11.98 ± 0.42% for small peptides. As shown in Figure 1b, when hydrolysis time, enzyme addition, and moisture content are kept constant, water-soluble protein and small-peptide contents increase and then decline with increasing temperature, with peak values at 35 °C (90.28 ± 2.22 mg/g and 11.92 ± 0.30%, respectively). According to Figure 1c, when enzyme addition, moisture content, and temperature were held constant, hydrolysis time showed a positive relationship with both water-soluble protein and small-peptide contents (*p* < 0.01). These values increased continuously and reached their maximum at 48 h. (95.14 ± 0.31 mg/g and 13.86 ± 0.11%, respectively). A rapid increase in peptide content between 24 and 48 h was observed, indicating efficient hydrolysis of SBM proteins during this interval. Notably, the peptide content did not decline at 72 h. This may be attributable to hydrolysis conditions near the protease’s operating optimum, which supported continued enzymatic action, and to sufficient substrate remaining in the semi-solid matrix, leading to sustained release and accumulation of low-molecular-weight peptides. As shown in Figure 1d, when hydrolysis time, enzyme addition, and temperature were fixed, water-soluble protein and small-peptide contents first increased and then decreased with increasing moisture levels (*p* < 0.05, *p* < 0.01). The highest water-soluble protein content was observed at 40% moisture (90.90 ± 2.92 mg/g), whereas the highest small-peptide content occurred at 30% moisture (12.74 ± 0.45%). This difference may reflect the complex effects of water content on enzymatic reactions in semi-solid systems. Appropriate water content is crucial for enzymatic reactions, as it promotes effective contact between the enzyme and substrate, thereby enhancing protein hydrolysis efficiency and influencing the production and accumulation of bioactive peptides [9,10].

### 3.2. Optimizing the Process Conditions for Enzymatic Digestion of Soybean Meal Through Response Surface Methodology

Based on the single-factor results, which identified small-peptide content as the response variable, clear interactions among the hydrolysis parameters were observed. Therefore, four factors—enzyme addition, temperature, time, and moisture content—were selected for response surface optimization. Multiple regression models were developed, and response surface plots were generated from the fitted equations to determine optimal hydrolysis conditions. This approach finalized the experimental design for the four-factor Box–Behnken response surface analysis. The factors and their levels are summarized in Table 1, and the experimental design and results are presented in Table 2.

$$\begin{array}{c} Small\;peptide=18.71-0.0796A+5.34B+0.7149C+0.4971D-0.9018AB\\ +\;1.02AC+0.9322AD-0.0952BC+1.05BD+0.2056CD-2.44A^{2}-4.24B^{2}\\ -0.9946C^{2}-3.93D^{2} \end{array}$$

The equation describes how the small-peptide content changes after enzymatic digestion of SBM and the factors that influence it. Response surface analysis yielded a model with *p* < 0.01, indicating high significance. With a correlation coefficient of R^2^ = 0.9701, the model explains 97% of the variation in the response. These results confirm the reliability of the experimental method, enabling analysis using the regression model rather than individual experimental data. However, the pure error was zero (Pure Error = 0), and the lack-of-fit *p*-value was neither estimable nor informative; therefore, model adequacy was assessed using overall model significance, goodness-of-fit metrics (R^2^, adjusted R^2^, predicted R^2^), and validation experiments.

### 3.3. Response Surface Result Analysis

**Figure 2:**
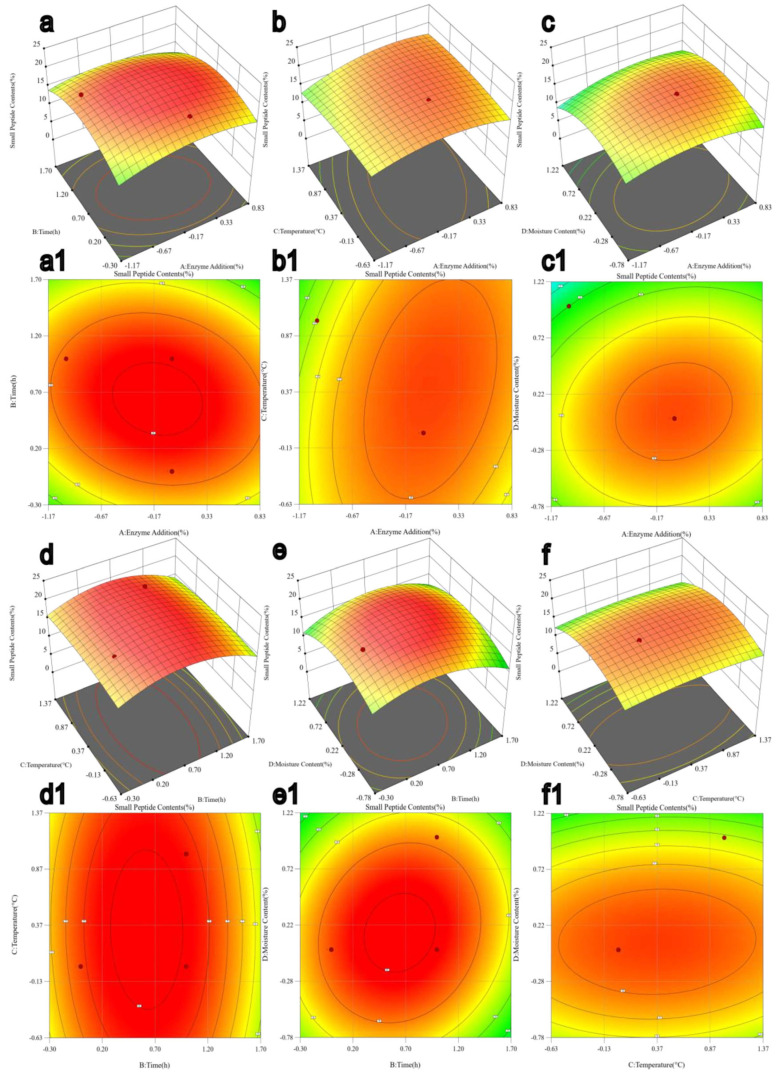
Three-dimensional response surface plot and contour map. Note: (**a**,**a1**) represent the 3D and contour maps of enzyme addition over time; (**b**,**b1**) show the 3D and contour maps of enzyme addition versus temperature; (**c,c1**) display the 3D and contour maps of enzyme addition versus moisture; (**d**,**d1**) illustrate the 3D and contour maps of time against temperature; (**e**,**e1**) depict the 3D and contour maps of time versus moisture; (**f**,**f1**) present the 3D and contour maps of temperature versus moisture. In a response surface plot, the 3D surface visualizes how the predicted response changes with two factors, while the color gradient encodes response magnitude to quickly highlight high/low (optimal) regions and the steepness of change.

The effects of enzyme addition (A), hydrolysis time (B), temperature (C), and moisture content (D) on small-peptide production were modeled in Design-Expert 13, and the corresponding contour plots and three-dimensional response surfaces are shown in Figure 2. The response surfaces were generated from the fitted quadratic regression model, and the statistical significance of model terms was evaluated by ANOVA (Table 3). As shown in Table 3, hydrolysis time (B) and the quadratic terms A^2^, B^2^, and D^2^ were statistically significant (*p* < 0.05), indicating pronounced curvature in the response within the tested ranges. In contrast, the linear terms A, C, and D and all interaction terms (AB, AC, AD, BC, BD, and CD) were not significant (*p* > 0.05). Therefore, Figure 2 is primarily used to visualize main-effect trends and quadratic curvature and to locate the optimum within the experimental domain, rather than to infer mechanistic interactions from non-significant terms. Consistent with this, most contour plots exhibit closed, near-elliptical patterns, supporting that the optimum lies within the investigated factor ranges.

Based on the regression model, the predicted optimal conditions for SBM enzymatic hydrolysis were 1.42% enzyme addition, 61.30 h hydrolysis time, 36.0 °C, and 34.90% moisture content, with a predicted small-peptide content of 18.712%. Considering practical feasibility, the operational conditions were adjusted to 1.45% enzyme addition, 62 h hydrolysis time, 36.5 °C, and 35% moisture content. Under these adjusted conditions, the experimentally obtained small-peptide content was 19.68%, close to the model prediction (relative deviation ≈ 5.17%), confirming the adequacy and practical applicability of the fitted model.

### 3.4. Scanning Electron Microscope Results

**Figure 3:**
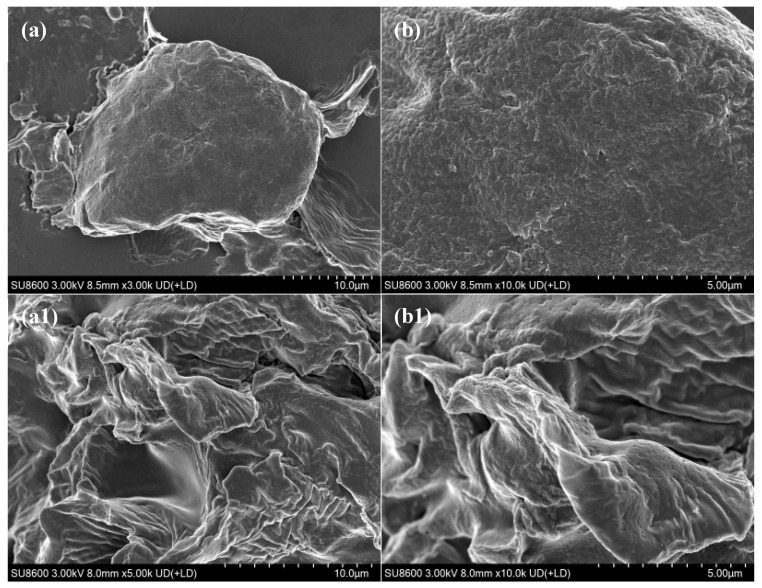
SEM images of soybean meal before and after enzymatic hydrolysis. Note: EHSBM: After enzymatic hydrolysis of soybean meal. (**a**): SEM image of SBM, magnification ×3000. Scale bar = 10.0 μm. (**b**): SEM image of SBM, magnification ×10,000. Scale bar = 5.0 μm. (**a1**): SEM image of EHSBM, magnification ×5000. Scale bar = 10.0 μm. (**b1**) SEM image of EHSBM, magnification ×10,000. Scale bar = 5.0 μm.

Figure 3a,b show that, prior to enzymatic hydrolysis, the SBM surface exhibited clearly defined plant cell structures with a dense, intact morphology typical of natural plant proteins. The tightly bound cell wall encapsulated internal nutrients, including proteins and lipids, with only minor surface debris present. This dense barrier restricted enzyme access and penetration, thereby limiting nutrient bioavailability. Following enzymatic hydrolysis, substantial structural disruption was observed on the SBM surface. As illustrated in Figure 3(a1,b1), the previously smooth, compact surface became eroded and fragmented, with numerous microscopic pores and fissures, many of which exhibited honeycomb- or sponge-like patterns.

### 3.5. Infrared Spectra of Proteins Before and After Enzymatic Digestion of Soybean Meal

**Figure 4:**
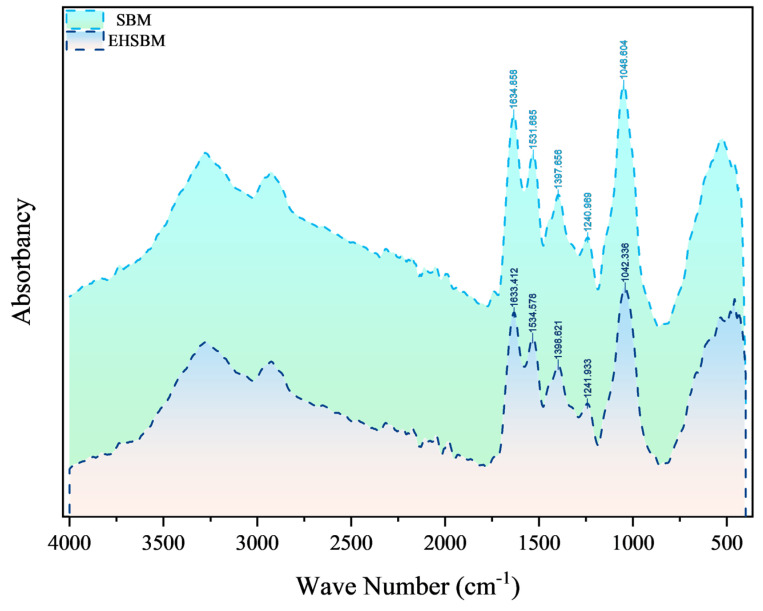
Infrared spectrum from Fourier transform.

The FTIR spectra of SBM before and after enzymatic hydrolysis are presented in Figure 4. In the amide I region, the major absorption peak shifted from 1633.412 to 1634.858 cm^−1^ following hydrolysis, indicating alterations in hydrogen bonding and changes in the microenvironment surrounding the carbonyl groups. This upward shift suggests an increase in flexible or disordered secondary structures, such as β-turns and random coils. In addition, the amide II peak shifted from 1534.578 to 1531.685 cm^−1^, reflecting modifications in the coupling of N–H bending and C–N stretching vibrations.

### 3.6. Variations in Protein Secondary Structure Before and After Enzymatic Hydrolysis of Soybean Meal

**Figure 5:**
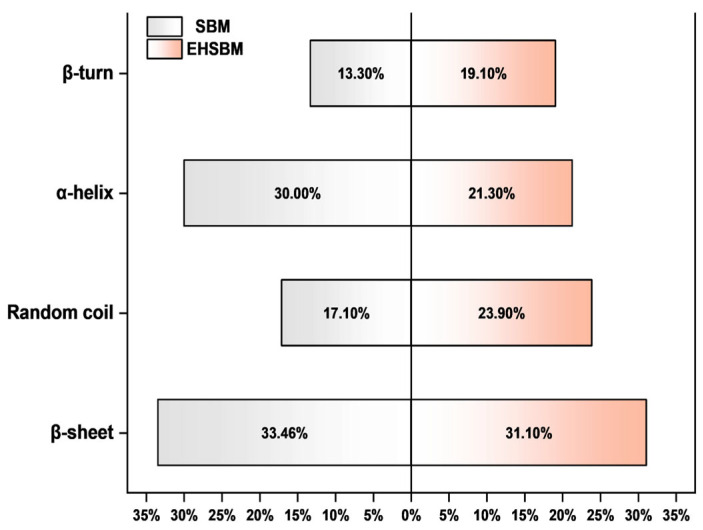
Comparison of protein secondary structure in soybean meal before and after enzymatic hydrolysis.

As shown in Table 4 and Figure 5, enzymatic hydrolysis markedly altered the secondary structure of SBM proteins. The amide I band shifted upward, while the amide II band shifted downward, indicating changes in carbonyl environments and hydrogen-bonding patterns. Overall, both peak area and peak height increased, and the area-to-height ratio also rose, suggesting increased exposure of peptide bonds and reorganization of the secondary structure. Structurally, the non-normalized α-helix band area increased (0.324 to 0.438), whereas its normalized proportion decreased (0.300 to 0.213); although the absolute α-helix content increased, which can be attributed to the more pronounced increases in β-turn and random-coil (and β-sheet) components after hydrolysis (Table 4). The β-sheet proportion remained relatively stable, whereas the proportions of β-turn and random-coil increased. Collectively, these results indicate a shift from more ordered conformations toward more flexible/disordered structures, consistent with the disruption of internal hydrogen bonding and loosening of the protein architecture.

### 3.7. Relative Molecular Weight Distribution

**Figure 6:**
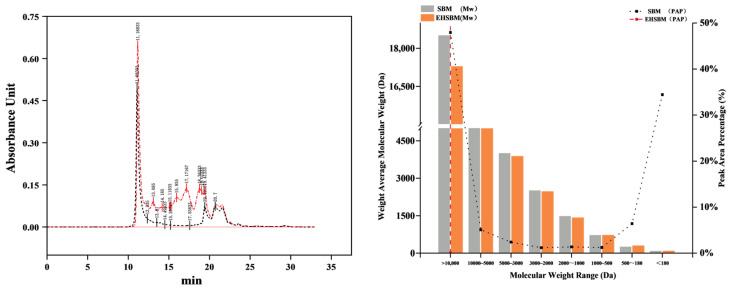
(**Left**): Relative molecular weight distribution. (**Right**): Weight-average molecular weight (Mw) and peak area percent (PAP).

The relative molecular-weight distributions of SBM and EHSBM are shown in Figure 6. Enzymatic hydrolysis markedly altered the molecular-weight profile, reducing the weight-average molecular weight from 9544 Da to 5817 Da—a 39% decrease. The proportion of high–molecular-weight proteins (>10,000 Da) decreased from 47.95% to 26.80%, while the abundance of low–molecular-weight peptides (180–2000 Da) increased substantially. Specifically, the proportions of peptides in the 2000–1000 Da, 1000–500 Da, and 500–180 Da ranges increased by approximately 6.3-, 9.2-, and 1.6-fold, respectively. These results demonstrate that enzymatic hydrolysis effectively cleaves large protein molecules into smaller peptide fragments that are generally considered more accessible for subsequent digestion and utilization.

### 3.8. Free Amino Acids

**Figure 7:**
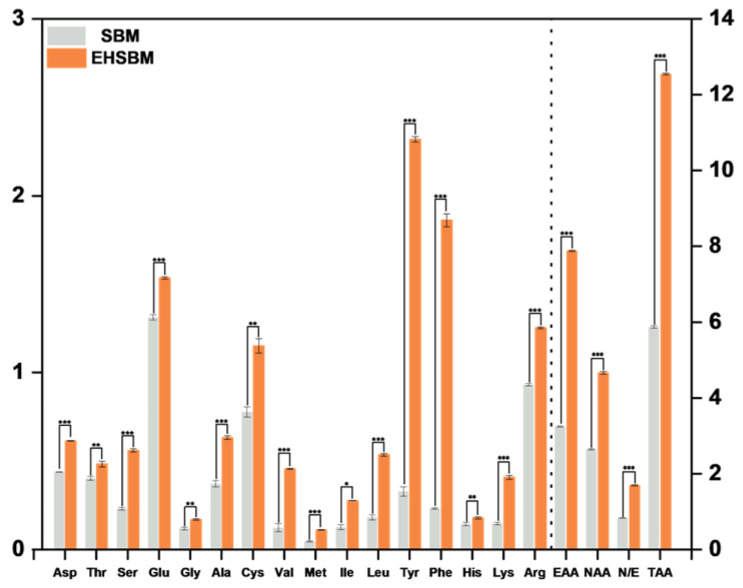
Free amino acid composition before and after enzymatic hydrolysis of soybean meal. Note: * *p* < 0.05, ** *p* < 0.01, and *** *p* < 0.001. The dashed line separates essential amino acids (EAAs) from non-essential amino acids (NEAAs); E/N, and total amino acids are shown on the right side of the dashed line.

Table 5 and Figure 7 show that enzymatic hydrolysis markedly altered the free amino acid profile of SBM. Total free amino acid content rose from 5.862 ± 0.030 mg/g before hydrolysis to 12.546 ± 0.022 mg/g after hydrolysis (*p* < 0.001), a 114.2% increase. This confirms that enzymatic hydrolysis effectively cleaves peptide bonds and releases free amino acids. Composition analysis revealed significant differences among individual amino acids after hydrolysis. Essential amino acids increased from 3.231 ± 0.016 mg/g to 7.883 ± 0.017 mg/g (*p* < 0.001), a 144.0% rise, markedly higher than the 77.2% increase in non-essential amino acids. Aromatic amino acids, such as tyrosine and phenylalanine, increased substantially (tyrosine: 0.325 ± 0.017 → 2.320 ± 0.016 mg/g; phenylalanine: 0.229 ± 0.004 → 1.862 ± 0.037 mg/g; both *p* < 0.001). Branched-chain amino acids (leucine, isoleucine, valine) and sulfur-containing amino acids (methionine, cysteine) also increased significantly (*p* < 0.001).

### 3.9. DPPH Free Radicals

**Figure 8:**
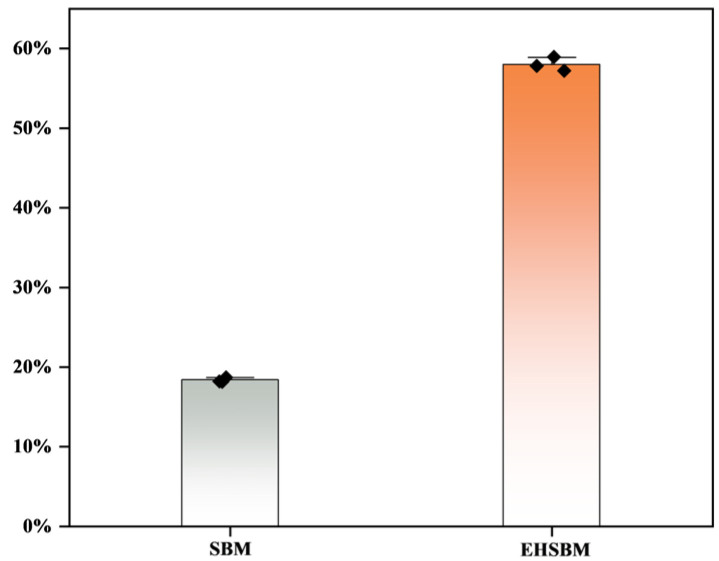
DPPH scavenging rate.

Figure 8 shows the DPPH radical-scavenging activity of SBM before and after enzymatic hydrolysis. The scavenging activity increased from 18.37% to 57.99%, an improvement of 39.62%. These results indicate that enzymatic hydrolysis substantially enhances the antioxidant capacity of SBM by improving its ability to quench DPPH free radicals.

## 4. Discussion

### 4.1. Improving the Soybean Meal Enzymatic Hydrolysis Process

The efficiency of enzymatic hydrolysis depends on appropriate enzyme preparations and precise control of processing parameters, including enzyme and substrate concentrations, temperature, time, and pH [11,12]. In practical applications, selecting suitable single or combined enzyme preparations requires consideration of the substrate’s chemical composition and antinutritional factors [13]. For SBM, combined protease hydrolysis is commonly used to break down large proteins into small peptides and free amino acids, thereby improving digestibility [4]. For cereal- or bran-based substrates, enzymes such as xylanase and β-mannanase are applied to degrade non-starch polysaccharides and disrupt cell wall structures [14]. Proper regulation of hydrolysis parameters—such as temperature, pH, substrate concentration, and reaction duration—is essential [15], as these factors directly affect hydrolysis efficiency and product stability. Previous studies have shown [16] that applying soybean meal–specific hydrolyzing enzymes increased the degree of hydrolysis to 48% and achieved a protein extraction rate of 81.2%. Similarly, Zhang et al. [17] reported that Alcalase treatment effectively degraded SBM proteins into small peptides. In their study, hydrolysis parameters including enzyme dosage, temperature, duration, and moisture content were optimized using single-factor experiments followed by Box–Behnken response surface methodology, identifying optimal conditions of 45% enzyme addition, 62 h, 36 °C, and 35% moisture. Under these conditions, small-peptide content reached 19%, representing a 16.31-fold increase. These findings demonstrate that combined protease preparations effectively hydrolyze high–molecular-weight soybean proteins, modify structural characteristics, and substantially increase small-peptide content.

### 4.2. Microstructure Analysis

Structural changes at the microlevel are closely associated with nutrient release and digestibility [18]. In SBM, the densely compacted surface encapsulates internal nutrients, such as proteins and lipids, limiting their contact with external enzymes and substantially reducing nutrient utilization [19,20]. Enzymatic hydrolysis disrupts and degrades this compact cell wall structure, generating a loose and porous matrix that exposes additional nutrients and enhances enzyme–substrate interactions, thereby improving protein hydrolysis and increasing nutritional value [21]. Wang et al. [22] reported that enzymatically hydrolyzed SBM exhibited a fibrous surface and a markedly loosened internal structure, which increased the contact area between enzymes and substrates. SEM analysis confirmed that enzymatic treatment effectively disrupted the compact cell wall, resulting in a porous, open structure. This transition from a “dense and closed” to a “loose and porous” state exposes previously inaccessible nutrients and facilitates subsequent hydrolysis. As a consequence, more protein molecules become available for enzymatic action, significantly enhancing the nutritional properties of SBM. This microstructural loosening is quantitatively consistent with improved enzymatic accessibility, as evidenced by the marked shift in molecular-weight distribution (Figure 6), with the weight-average molecular weight decreasing from 9544 Da to 5817 Da and the proportion of >10,000 Da fractions declining from 47.95% to 26.80%, accompanied by substantial enrichment of low-molecular-weight peptides (180–2000 Da).

### 4.3. Analysis of FTIR

Infrared spectroscopy is a widely used analytical technique for characterizing molecular structures. They are characterized by their sensitivity to chemical composition and molecular vibrations [23]. Each functional group exhibits characteristic absorption peaks [24], with approximate positions determined by vibrational mass and bond type (single, double, or triple). The precise locations of these peaks are further influenced by the electron-withdrawing or electron-donating effects of the surrounding chemical environment and by vibrational coupling.

In proteins, the major infrared features arise from peptide-bond vibrations, commonly called the amide bands. Among them, the amide I (1600–1700 cm^−1^) and amide II (1480–1580 cm^−1^) bands are the most informative for secondary-structure analysis, as they are highly sensitive to α-helix, β-sheet, β-turn, and random-coil conformations. Approximately 80% of the amide I signal originates from C=O stretching vibrations, while the amide II band primarily reflects N–H bending and C–N stretching. These two bands are therefore widely used to evaluate protein structural composition and conformational changes [25]. Previous studies have shown that hydrolysis of SBM with an alkaline protease results in noticeable shifts in the amide I and amide II bands, attributed to disruption of hydrogen bonds and unfolding of helical structures [26]. In this experiment, the amide I band shifted upward from 1633.412 cm^−1^ to 1634.858 cm^−1^, while the amide II band shifted from 1534.578 cm^−1^ to 1531.685 cm^−1^ following enzymatic hydrolysis of SBM. Structurally, the relative proportion of α-helix decreased, whereas β-sheet, β-turn, and random-coil structures increased after hydrolysis. Mechanistically, these spectral shifts and secondary-structure redistribution likely reflect hydrolysis-driven cleavage of peptide chains and the accompanying reorganization of the hydrogen-bond network. Proteolysis preferentially targets accessible, flexible regions, weakening intramolecular hydrogen bonds that stabilize ordered conformations (especially α-helices) and promoting partial unfolding with increased backbone exposure. Consequently, the protein population shifts toward more flexible, disordered conformations, as evidenced by increased β-turn and random-coil components, which are commonly associated with shorter, less-ordered peptide segments. The comparatively limited change in β-sheet proportion may indicate that β-sheet-rich domains in soybean storage proteins are relatively resistant within the tested ranges and/or that unfolding and conformational reorganization occur concurrently, resulting in a small net change in the normalized β-sheet fraction. These changes are likely attributable to the disruption of internal hydrogen bonds, which converts the compact secondary structure of SBM into a looser, more disordered conformation that may facilitate subsequent enzymatic degradation and digestion, thereby making the hydrolysates potentially more bioavailable. These findings align with the microstructural observations obtained from SEM [27].

### 4.4. Analysis of Protein Relative Molecular Mass Distribution

Protein molecular weight distribution is an important indicator of the effectiveness of enzymatic hydrolysis [15]. During hydrolysis, complex high–molecular-weight proteins are cleaved into smaller peptides that are more readily absorbed, as these low–molecular-weight fragments exhibit faster and more efficient uptake than intact proteins [28]. In this study, molecular weight analysis before and after enzymatic treatment demonstrated clear protease activity. The hydrolysis process markedly altered the protein size profile of SBM, reducing the weight-average molecular mass by 39%, decreasing the proportion of high–molecular-weight proteins, and increasing the abundance of smaller peptide fragments. This substantial reduction in high–molecular-weight fractions indicates that the protease effectively cleaved large protein chains into smaller units, corroborating the structural changes observed in the SEM and FTIR analyses.

### 4.5. Analysis of Free Amino Acids

Among plant-derived protein sources, soybean is considered one of the most biologically valuable due to its amino acid profile, which closely resembles that of animal proteins, particularly in terms of essential amino acid composition [29]. Studies have shown that soybean protein content exceeds that of hemp seeds and exhibits a more favorable amino acid balance [30]. SBM, a major by-product of soybean processing, is similarly rich in essential amino acids, particularly lysine, which is typically limited in cereal grains [31]. Enzymatic hydrolysis of SBM using enzymes produced by *Aspergillus oryzae* has been reported to significantly increase glutamic and aspartic acid levels [32]. Amino acids, as the fundamental building blocks of proteins, directly affect the composition and quality of animal tissues and play indispensable roles in growth, development, reproduction, immunity, and other physiological processes [33]. Therefore, dietary protein requirements are essentially determined by the need for specific amino acids—particularly essential amino acids that animals cannot synthesize adequately and must obtain from feed [34]. Deficiencies in protein or amino acids impair immune function and increase susceptibility to infectious diseases in both animals and humans [35,36]. In this study, enzymatic hydrolysis markedly increased the free amino acid content of SBM. Essential amino acids nearly doubled, and aromatic as well as branched-chain amino acids also showed pronounced increases. Furthermore, DPPH radical-scavenging assays showed a substantial increase in antioxidant activity after enzymatic treatment, consistent with the findings of Da et al. [37]. This increase may be associated with the concurrent release of low-molecular-weight peptides and free amino acids during hydrolysis. These results indicate that enzymatic hydrolysis effectively disrupts the dense structure of SBM, releases previously inaccessible components, partially converts them into free amino acids, potentially increases their availability, and enhances the in vitro radical-scavenging activity (DPPH) of the hydrolysates.

## 5. Conclusions

The microstructure and physicochemical properties of SBM were substantially changed under optimal enzymatic hydrolysis conditions. The enzymatic process effectively disrupted the surface integrity and compact structure of SBM, generating honeycomb- or sponge-like porous formations. Correspondingly, the protein secondary structure shifted from an ordered conformation to a looser and more disordered state, exposing additional peptide bonds. After hydrolysis, the average molecular weight decreased markedly from 9544 Da to 5817 Da (a 39% reduction), accompanied by a substantial increase in small peptide fragments within the 180–2000 Da range. Component analysis further showed that the total free amino acid content increased greatly from 5.862 mg/g to 12.546 mg/g (114.2%), with essential amino acids rising by 199.6%. In addition, the in vitro DPPH radical-scavenging activity of the hydrolysates increased by 39.62%. Overall, these results demonstrate that enzymatic hydrolysis can effectively remodel the structural organization of SBM and potentially increase the availability of peptides and free amino acids, providing quantitative evidence to support further exploration of SBM-derived protein/peptide ingredients.

## Figures and Tables

**Table 1:** Factors and levels of the Box–Behnken test.

Level	Factor			
	Enzyme Addition (%)	Temperature (°C)	Time (h)	Moisture (%)
−1	1	30	24	30
0	1.5	35	48	35
1	2	40	72	40

**Table 2:** Response surface design and test results.

No.	Enzyme Addition (%)	Moisture (%)	Time (h)	Temperature (°C)	Small Peptide (%)
1	0	0	0	0	18.80
2	0	1	1	0	19.680
3	0	1	1	0	19.68
4	0	−1	−1	−1	5.58
5	0	0	0	0	18.80
6	0	0	1	−1	13.86
7	0	0	0	0	18.80
8	1	0	0	0	17.36
9	0	0	1	1	13.58
10	0	0	−1	1	13.10
11	−1	0	−1	0	15.42
12	0	1	−1	0	18.37
13	0	1	0	1	17.04
14	−1	1	0	0	19.63
15	1	0	−1	0	13.60
16	1	−1	0	0	5.55
17	0	0	0	0	18.80
18	−1	−1	1	0	5.19
19	−1	0	0	−1	11.37
20	0	−1	−1	0	6.11
21	0	0	0	0	18.80
22	0	−1	0	1	5.68
23	1	1	0	0	14.96
24	1	0	0	1	15.07
25	0	1	0	0	19.67
26	1	0	1	0	17.66
27	−1	−1	0	0	6.09
28	−1	0	1	0	14.88
29	−1	0	0	1	11.88

**Table 3:** Variance analysis and significance tests.

Source	Sum of Squares	df	Mean Square	F-Value	*p*	
Model	703.59	14	50.26	32.50	<0.0001	**
A	0.0709	1	0.0709	0.0459	0.8335	
B	270.48	1	270.48	174.90	<0.0001	**
C	5.69	1	5.69	3.68	0.0756	
D	1.38	1	1.38	0.8911	0.3612	
AB	3.52	1	3.52	2.28	0.1537	
AC	4.51	1	4.51	2.92	0.1097	
AD	2.01	1	2.01	1.30	0.2735	
BC	0.0382	1	0.0382	0.0247	0.8773	
BD	2.16	1	2.16	1.40	0.2567	
CD	0.1349	1	0.1349	0.0872	0.7720	
A^2^	34.97	1	34.97	22.61	0.0003	
B^2^	111.30	1	111.30	71.97	<0.0001	**
C^2^	6.33	1	6.33	4.10	0.0625	
D^2^	62.53	1	62.53	40.44	<0.0001	**
Residual	21.65	14	1.55			
Lack of Fit	21.65	9	2.41			
Pure Error	0.0000	5	0.0000			
Total	725.24	28				

** indicates extreme significance, at *p* ≤ 0.01.

**Table 4:** Secondary structural parameters before and after digestion of soybean meal.

Items	Group		SEM
	SBM	EHSBM	
Initial analysis of the amide I region	1580.065–1708.877	1583.292–1691.659	
	1.079	2.055	0.488
Amide I center	1633.412	1634.858	
The amide I region reaches a high peak	0.065	0.096	0.031
Initial analysis of the amide II region	1481.530–1580.065	1484.772–1583.292	
Peak area of amide II	0.448	0.569	0.061
Amide II zone center	1534.578	1531.685	
The amide II region reaches a high peak	0.034	0.035	0.001
Peak area of amide I region/Peak area of amide II region	2.408	3.612	0.602
Peak height in the amide I region/Peak height in the amide II region	1.912	2.743	0.416
β-sheet area	0.361	0.638	0.014
Proportion of β-sheet peak area	0.335	0.311	0.001
Random coil area	0.184	0.492	0.154
Proportion of random coil peak area	0.171	0.239	0.034
α-helix area	0.324	0.438	0.057
Proportion of α-helix peak area	0.300	0.213	0.044
β-turn area	0.144	0.392	0.124
Proportion of β-turn peak area	0.133	0.191	0.029
β-turn area/α-helix area	0.111	0.146	0.018

**Table 5:** Effects of enzymatic hydrolysis of soybean meal on amino acid content (*n* = 3).

Items	SBM	EHSBM	*p*-Value
Aspartic acid	0.435 ± 0.001	0.614 ± 0.002	<0.0001 ***
Threonine	0.399 ± 0.011	0.483 ± 0.016	0.0043 **
Serine	0.228 ± 0.008	0.560 ± 0.009	<0.0001 ***
Glutamic acid	1.311 ± 0.015	1.536 ± 0.006	0.0001 ***
Glycine	0.115 ± 0.007	0.169 ± 0.004	0.0002 **
Alanine	0.368 ± 0.018	0.632 ± 0.010	0.0001 ***
Cystine	0.775 ± 0.029	1.152 ± 0.041	0.0003 **
Valine	0.120 ± 0.023	0.456 ± 0.004	<0.0001 ***
Methionine	0.043 ± 0.004	0.111 ± 0.002	<0.0001 ***
Isoleucine	0.123 ± 0.014	0.277 ± 0.001	0.0027 *
Leucine	0.178 ± 0.014	0.535 ± 0.008	<0.0001 ***
Tyrosine	0.325 ± 0.026	2.320 ± 0.016	<0.0001 ***
Phenylalanine	0.229 ± 0.004	1.862 ± 0.037	<0.0001 ***
Histidine	0.140 ± 0.009	0.178 ± 0.006	0.0010 **
Lysine	0.143 ± 0.007	0.408 ± 0.011	<0.0001 ***
Arginine	0.929 ± 0.007	1.253 ± 0.005	<0.0001 ***
Essential Amino Acid	3.231 ± 0.016	7.883 ± 0.017	<0.0001 ***
Non-Essential Amino Acid	2.631 ± 0.014	4.663 ± 0.038	<0.0001 ***
E/N	0.814 ± 0.001	1.691 ± 0.017	<0.0001 ***
Total	5.862 ± 0.030	12.546 ± 0.022	<0.0001 ***

Note: *** indicates extremely significant differences (*p* ≤ 0.001); ** indicates highly significant differences (0.001 < *p* ≤ 0.01); * indicates significant differences (0.01 < *p* ≤ 0.05). E/N: Essential Amino Acid/ Non-Essential Amino Acid.

## Data Availability

The original contributions presented in this study are included in the article. Further inquiries can be directed to the corresponding author.

## Abbreviations

The following abbreviations are used in this manuscript:

SBM	Soybean meal
EHSBM	Enzymatically hydrolyzed soybean meal
RSM	Response surface methodology
BBD	Box–Behnken design
SEM	Scanning electron microscopy
FTIR	Fourier transform infrared spectroscopy
